# Prevalence and associated risk factors of soil-transmitted helminth infections in Kandahar, Afghanistan

**DOI:** 10.1186/s12879-022-07336-z

**Published:** 2022-04-11

**Authors:** Bilal Ahmad Rahimi, Bashir Ahmad Mahboobi, Mohammad Hashim Wafa, Mohammad Sediq Sahrai, Muhammad Haroon Stanikzai, Walter R. Taylor

**Affiliations:** 1 Department of Pediatrics, Faculty of Medicine, Kandahar University, Beside Aino Mena Town, District 10, Durahi, Kandahar, Afghanistan; 2 Head of Research Unit, Faculty of Medicine, Kandahar University, Kandahar, Afghanistan; 3 Department of Psychiatry, Faculty of Medicine, Kandahar University, Kandahar, Afghanistan; 4 Department of Internal Medicine, Faculty of Medicine, Kandahar University, Kandahar, Afghanistan; 5 Department of Public Health, Faculty of Medicine, Kandahar University, Kandahar, Afghanistan; 6 Mahidol Oxford Tropical Medicine Clinical Research Unit (MORU), Mahidol University, Bangkok, Thailand

**Keywords:** Prevalence, Soil-transmitted helminth, Children, Risk factor, Afghanistan

## Abstract

**Background:**

Soil-transmitted helminth (STH) infections are still a major health problem, especially in resource-limited countries. The community-based prevalence of STH is unknown in Afghanistan. Main objectives of this study were to estimate the prevalence and associated factors of STH among children in Daman district of Kandahar province in Afghanistan.

**Methods:**

This was a community-based cross-sectional study, with data collected during five months (June–October, 2020) from children living in five villages of Daman district in Kandahar, Afghanistan. All the stool samples were examined by saline wet mount method. Data were analyzed by using descriptive statistics, Chi square test, and multivariate logistic regression.

**Results:**

A total of 1426 children were studied, with majority (61.8%) of males and the mean age of 6.3 years. The overall prevalence of any intestinal parasitic infection was 39.8%. The overall prevalence of STH infection was 22.7%, with *Ascaris lumbricoides* (18.7%) as the most prevalent STH species, followed by hookworm (7.5%) and *Trichuris trichiura* (1.4%). Single, double, and triple STH infections were present in 14.9%, 7.2%, and 0.6% of the children, respectively. Multivariate logistic regression revealed that not washing hands after defecating/before eating (AOR 7.0, 95% CI 3.4–14.0), living in mud house (AOR 3.5, 95% CI 1.6–7.4), walking barefoot (AOR 2.2, 95% CI 1.6–3.1), living in overcrowded house (AOR 1.6, 95% CI 1.1–2.3), and practicing open defecation (AOR 1.4, 95% CI 1.1–2.0) as the risk factors associated with the predisposition of rural children for getting STH in Daman district of Afghanistan.

**Conclusions:**

Prevalence of STH is high among children of Daman district in Afghanistan. Most of the risk factors are related to poverty, decreased sanitation, and improper hygiene. Improvement of socioeconomic status, sanitation, and health education to promote public awareness about health and hygiene together with periodic mass deworming programs are better strategies for the control of STH infections in Afghanistan. Also, government and international donor agencies in Afghanistan should help in improving socio-economic status of the rural areas through provision of basic facilities such as piped water, electricity, good housing, and proper toilets.

## Background

Soil-transmitted helminths (STH) belong to a group of neglected tropical diseases, which mostly affect the tropical and subtropical regions. STH affect more than 1.5 billion people (24% of the world population) in Africa, Asia, and Latin America [1–3]. Approximately 267 million preschool-age and 568 million school-age children live in areas where STH transmission is very high [1].

Globally, the most prevalent STH is *Ascaris lumbricoides* (*A. lumbricoides*) (infecting approximately 1.2 billion people), followed by *Trichuris trichiura* (*T. trichiura*) (infecting nearly 795 million people) and hookworm (*Ancylostoma duodenale* and *Necator americanus*) which infects approximately 740 million people [4, 5]. School age children are at high risk of being infected with STH. This could be due to the reason that these children are more exposed to contaminated soil when they play, walk barefoot, eat soil, and do not practice good personal hygiene [6]. STH infections may cause many pathologic conditions [7], micronutrient impairment, and iron deficiency anemia [8–10]. Deworming campaigns in different countries of the world have shown to improve nutritional status, cognition, and school performance in school-age children [11–13].

Afghanistan has been suffering from military and civil conflict for the last four decades which, combined with natural disasters, has extremely weakened economic development [14]. The community-based prevalence of STH is unknown in Afghanistan. Diagnosis is mostly made on clinical basis without any laboratory confirmation. The insecurity and shortage of medical staff at all levels of the healthcare system hinders the implementation of epidemiological surveillance [15]. Healthcare system in Afghanistan is mostly dependent of international humanitarian aid [16]. The risk of parasitic diseases is estimated to be very high in Afghanistan [17].

Unfortunately, to our knowledge, there is currently no published data from Afghanistan to find the community-based prevalence and risk factors of STH in children. In 2003, workers with the World Health Organization (WHO) conducted a school-based survey with the help of Afghan ministry of public health and Afghan ministry of education. They collected fecal samples from 1,001 school children in four provinces of Afghanistan (Kabul, Kandahar, Nangarhar, and Farah) for soil-transmitted helminths (STH). This survey revealed that 47% of the school children were infected with STH, with predominant (41%) species of *A. lumbricoides* [18]. In 2017, a follow-up survey was conducted among school children aged 8–10 years to provide an update on STH epidemiology in Kabul, Balkh, Herat, Nangarhar, and Kandahar provinces of Afghanistan. In this survey, 26.6% of the children had at least one STH infection, with predominance of *A. lumbricoides* (25.7%) [19].In another hospital-based study in Afghanistan, 548 fecal samples were collected from the patients (both children and adults) with internal complaints who were admitted in two hospitals of Ghazni and Parwan provinces. Intestinal helminths were present in 144/386 (37.3%) of patients from Ghazni and Parwan provinces, respectively [15]. Main objectives of this community-based study were to estimate the prevalence of STH in children and also find the associated factors that predispose the rural children for getting STH in Daman district of Afghanistan.

## Methods

### Study design and study area

**Fig. 1:**
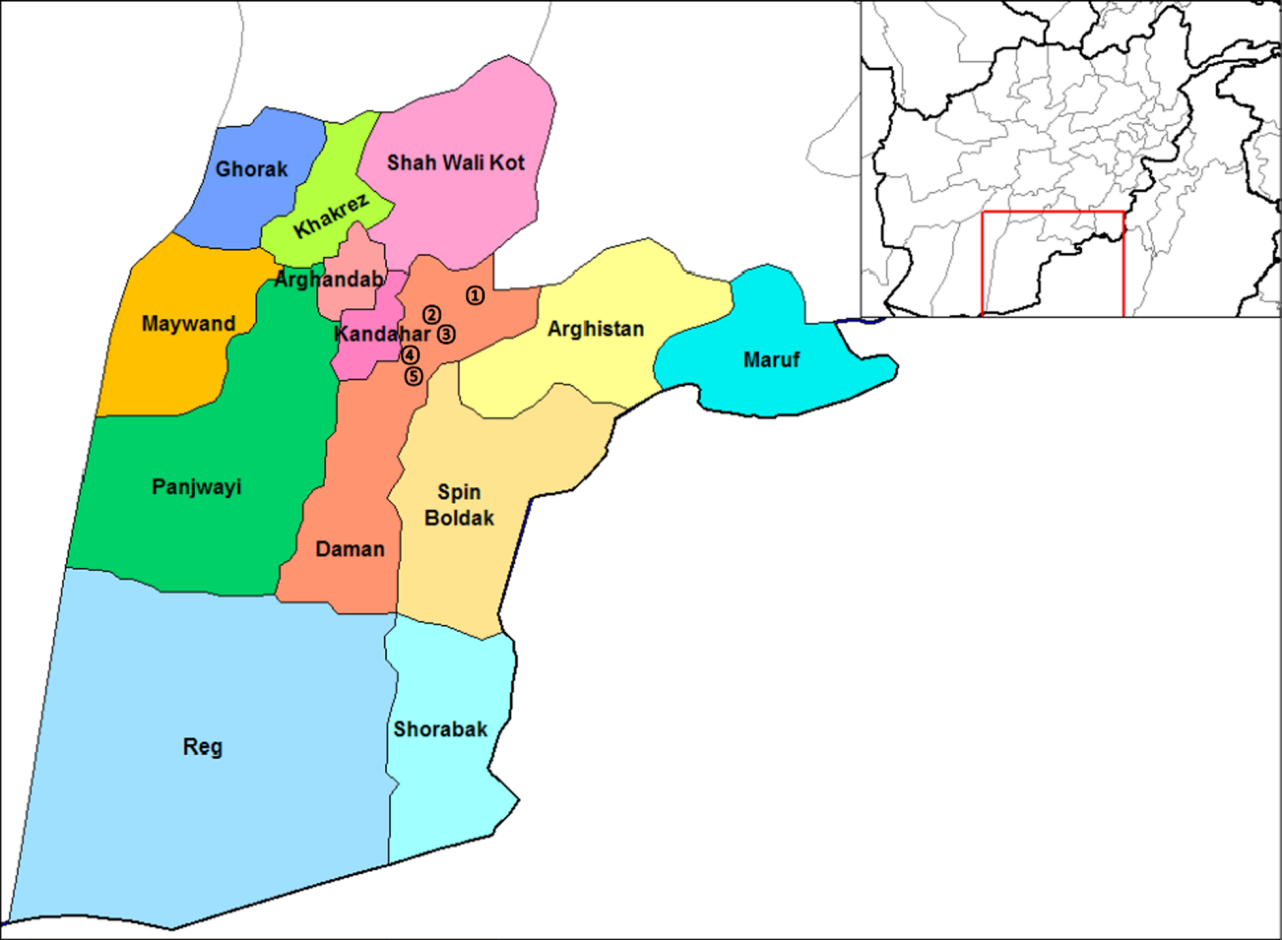
Map of Daman district in Kandahar province, Afghanistan. (1, Azam Kala village; 2, Ghara Kalai village; 3, Mandisar village; 4, Mula Abdullah village; 5, Khoshab village.) Source: https://commons.wikimedia.org/wiki/File:Kandahar_districts.png

This was a community-based cross-sectional study, conducted during five-month-period (June–October, 2020). All the villages of Daman district with ≥ 50 houses each were selected for randomization using lottery method. After randomization, five villages (Azam Kala, Ghara Kalai, Mandisar, Mula Abdullah, and Khoshab) were selected for the study. Southern part of Daman district is covered with desert with nearly no inhabitants, while the northern part has cultivated lands and villages. Two seasonal rivers (Tarnak river and Arghistan river) and one running water canal from Arghandab river are flowing through the northern part of Daman district (Fig. 1). For stool samples examination, 3 health facilities were selected (Mandisar comprehensive health center, Khoshab basic health center, and Azam Kala basic health center) with every health facility within one kilometer distance from their nearby selected villages.

### Study population

The study population was composed of children with age < 15 years and permanent residents of Daman district in Kandahar province. All those children were excluded from the study who received any anti-helminthic treatment in the previous three months before the commencement of the study, having chronic diseases, not able to provide stool samples, or their parents/guardians refuse to participate in the study. A total of 1595 children were included to this study. Among these children, parents/guardians of 77 (4.8%) children refused to take part in the study while 92 (5.8%) failed to submit their fecal samples. So, data was collected from 1426 children.

### Ethical considerations

Written informed consents were taken from parents or guardians of all the participants prior to the study. From children < 15 years old but old enough to answer were given the assent forms for the participation. Information of the participants will not be disclosed. Ethical approval was taken from Kandahar University Ethics Committee (code number KDRU-EC-2020.91). For data collection, only patients' initials were used. Prior to entering into the computer for analysis, the collected data was coded and de-identified.

### Sample collection and laboratory procedures

Systematic random sampling was used for selecting the households while lottery method was used for selecting only one child from each household. A questionnaire was utilized in Pashto language with questions regarding general characteristics, economic status, general sanitation and environmental conditions, and laboratory examination. Data were collected/recorded on paper forms by experienced and trained investigators. Prior to data collection, meetings were conducted in the selected villages. A short briefing was given, during which the objectives and method of the study were clearly informed to the village elders and heads of the families, and prospective participants.

The case record form and other materials were pretested before the actual data collection. Persons responsible for data collection were well trained on how to conduct the interview and how to collect the stool samples. One day before sample collection, the caretakers of the children who agreed to participate in the study were provided with clean pre-labeled capped plastic container for stool collection along with instruction on correct placement of the stool into the containers. All the caretakers were instructed to collect the stool samples of the children from 8–11 am. As the assigned health facility were near to their villages, the assigned study volunteers in the villages would bring the stool samples immediately within 15 min to the laboratories of health facilities using motorbikes. In the laboratory, for the detection of intestinal parasites, saline wet mount method of stool examination was used. Although Kato–Katz technique is the preferred stool examination method for STH studies, but saline wet mount method was used in our study as this is the only available method in the public laboratories throughout Afghanistan. Stool examinations were performed by experienced medical laboratory technicians. All the stool samples were examined within 30 min after sample collection. So, there was no need for preservatives to store the samples. Some stool samples were randomly selected for quality control and examined by another experienced laboratory technician who was blinded for the previous test result. Also, to minimize the errors, data was double entered.

### Data analysis

The data were entered into Microsoft Excel, cleaned, and imported to SPSS version 22 (Chicago, IL, USA) for statistical analysis. Descriptive analysis including frequency, mean, standard deviation (SD), and range was used to summarize demographic characteristics. Frequency and percentage were used to summarize categorical variables. Chi-square test (using crude odds ratio [COR]) was performed to assess the binary association between various categorical variables. All variables that were statistically significant in univariate analyses were assessed for independence in a multivariate logistic regression (using adjusted odds ratio [AOR]) to determine the factors associated with the predisposition of rural children for getting STH. A *P*-value of < 0.05 was statistically significant.

## Results

As 169/1595 (10.6%) of the participants failed to submit their fecal samples, so the response rate of the study participants was 89.4% and the data was collected from 1426 children. Among 1426 children, 288 (20.2%) were the residents of Mandisar village, 287 (20.1%) Ghara Kalai village, 285 (20.0%) Khoshab village, 284 (19.9%) Mula Abdullah village, and 282 (19.8%) were the residents of Azam Kala village.

Mean (SD) age of these children was 6.3 (3.2) years with 331/1426 (23.2%) having less than five years of age. Male gender predominated (881/1426 [61.8%]) with 1285/1426 (90.1%) belonging to families with monthly income < 2500 Afghanis (< 30 USD). Nearly all (1387/1426 [97.3%]) mothers of these children were illiterate, while most (1161/1426 [81.4%]) of the guardians of their family were farmers (Table 1).

**Table 1:** Socio-demographic and other characteristics of the study participants

Variable	Number (n = 1426)	Percentage (%)
Age (years)		
< 5	331	23.2
5–10	724	50.8
> 10	371	26
Gender		
Male	881	61.8
Female	545	38.2
Family monthly income (in Afghanis)		
< 2500 (< 30 USD)	1285	90.1
2500–20,000 (30–250 USD)	107	7.5
> 20,000 (> 250 USD)	34	2.4
Family size		
< 5 people	330	23.1
≥ 5 people	1096	76.9
Mother’s education		
Primary	35	2.4
Secondary	4	0.3
Bachelor	0	0
Uneducated	1387	97.3
Father’s (guardian of family) occupation		
Farmer	1161	81.4
Jobless	194	13.6
Shopkeeper	46	3.2
Driver	25	1.8
House construction		
Mud	1314	92.1
Concrete	112	7.9
Source of drinking water		
Boreholes	526	36.9
Irrigation canal	687	48.2
Open dug wells	213	14.9
Use of toilet		
Pit toilets	364	25.5
Septic tank toilets	45	3.2
Open defecation	1017	71.3
Washing hands after defecating/before eating		
Yes	474	33.2
No	952	66.8
Walking barefoot		
Yes	962	67.5
No	464	32.5
Finger nail status		
Untrimmed	1138	79.8
Trimmed	288	20.2
Habit of nail biting		
Yes	215	15.1
No	1211	84.9
Consumption of raw vegetables		
Yes	1250	87.7
No	176	12.3
Habit of eating soil		
Yes	50	3.5
No	1376	96.5

*USD* United States Dollar

The overall prevalence of STH infection was 22.7% (324/1426 children). *A. lumbricoides* (18.7%, 267/1426) was the most prevalent STH species, followed by hookworm (7.5%, 107/1426), and *T. trichiura* (1.4%, 20/1426). Prevalence of intestinal protozoa infection was 17.9% (255/1426) while prevalence of overall any intestinal parasitic infection was 39.8% (567/1426). *Giardia intestinalis* was the most prevalent intestinal protozoa with a prevalence of 13.9%. Among the STH infected patients, single infection, double infection, and triple infections were present in 213/1426 (14.9%), 103/1426 (7.2%), and 8/1426 (0.6%) of the children, respectively (Table 2).

**Table 2:** Species of intestinal parasitic infection among rural children in Daman district

Intestinal parasitic infection	Number (n = 1426)	Prevalence (%)
Overall any intestinal parasitic infection	567	39.8
Monoparasitism	409	28.7
Polyparasitism	158	11.1
Overall any STH infection	324	22.7
Single STH infection	213	14.9
Double STH infection	103	7.2
Triple STH infection	8	0.6
Overall any intestinal protozoa infection	255	17.9
STH		
*Ascaris lumbricoides*	267	18.7
Hookworm	107	7.5
*Trichuris trichiura*	20	1.4
Protozoa		
*Giardia intestinalis*	198	13.9
Entamoeba spp.	123	8.6
Other intestinal parasites		
*Hymenolepis nana*	194	13.6
Taenia spp.	84	5.9
*Enterobius vermicularis*	37	2.6

*STH* Soil-transmitted helminth

In Chi-square test, statistically significant variables responsible for increased STH infection were children from overcrowded families (COR 1.6, 95% CI [confidence interval] 1.2–2.1, and *p*-value 0.001), living in mud house (COR 4.1, 95% CI 2.0–8.5, and *p*-value < 0.001), practicing open defecation (COR 1.6, 95% CI 1.2–2.2, and *p*-value 0.001), not washing hands after defecation and before eating (COR 3.3, 95% CI 2.4–4.6, and *p*-value < 0.001), walking barefoot (COR 2.5, 95% CI 1.9–3.5, and *p*-value < 0.001), having untrimmed finger nails (COR 1.9, 95% CI 1.4–2.7, and *p*-value < 0.001), habit of nail biting (COR 1.5, 95% CI 1.1–2.1, and *p*-value 0.012), and consuming raw vegetables (COR 1.6, 95% CI 1.0–2.4, and *p*-value 0.035) (Table 3).

**Table 3:** Chi-square test of the risk factors associated with increased STH in children

Variable	Total, n(n = 1426)	STH infection present	COR	95% CI	*P*-value
		n (%) (n = 324)			
Age (years)				0.8–1.5	0.386
< 5	331	81 (24.5)	1.1		
≥ 5	1095	243 (22.2)	1		
Gender				0.8–1.4	0.501
Male	881	195 (22.1)	1		
Female	545	129 (23.7)	1.1		
Family monthly income (in Afghanis)				1.0–2.5	0.056
< 2,500 (< 30 USD)	1285	301 (23.4)	1.6		
≥ 2,500 (≥ 30 USD)	141	23 (16.3)	1		
Family size				1.2–2.1	0.001
< 5 people	330	98 (29.7)	1.6		
≥ 5 people	1096	226 (20.6)	1		
Mother’s education				0.2–1.3	0.135
Educated	39	5 (12.8)	1		
Uneducated	1387	319 (23.0)	0.5		
Father’s (guardian of family) occupation				0.9–1.7	0.206
Farmer	1161	256 (22.0)	1		
Non-farmer	265	68 (25.7)	1.2		
House construction				2.0–8.5	< 0.001
Mud	1314	316 (24.0)	4.1		
Concrete	112	8 (7.1)	1		
Source of drinking water				0.5–0.8	< 0.001
Safe	526	91 (17.3)	1		
Unsafe	900	233 (25.9)	0.6		
Use of toilet				1.2–2.2	0.001
Yes	409	69 (16.9)	1		
Open defecation	1017	255 (25.1)	1.6		
Washing hands after defecating/before eating				2.4–4.6	< 0.001
Yes	474	51 (10.8)	1		
No	952	273 (28.7)	3.3		
Walking barefoot				1.9–3.5	< 0.001
Yes	962	264 (27.4)	2.5		
No	464	60 (12.9)	1		
Finger nails status				1.4–2.7	< 0.001
Trimmed	288	42 (14.6)	1		
Untrimmed	1138	282 (24.8)	1.9		
Habit of nail biting				1.1–2.1	0.012
Yes	215	63 (29.3)	1.5		
No	1211	261 (21.6)	1		
Consumption of raw vegetables				1.0–2.4	0.035
Yes	1250	295 (23.6)	1.6		
No	176	29 (16.5)	1		
Habit of eating soil				0.6–2.1	0.826
Yes	50	12 (24.0)	1.1		
No	1376	312 (22.7)	1		
Domestic animals present at home				0.4–0.8	0.002
Yes	1196	290 (24.2)	0.5		
No	230	34 (14.8)	1		

CI, confidence interval; COR, crude odds ratio; STH, Soil-transmitted helminth; USD, United States Dollar

Multivariate logistic regression of the above-mentioned statistically significant variables revealed that not washing hands after defecating/before eating (AOR 7.0, 95% CI 3.4–14.0, and *p*-value < 0.001), living in mud house (AOR 3.5, 95% CI 1.6–7.4, and *p*-value 0.001), walking barefoot (AOR 2.2, 95% CI 1.6–3.1, and *p*-value < 0.001), living in overcrowded house (AOR 1.6, 95% CI 1.1–2.3, and *p*-value 0.023), and practicing open defecation (AOR 1.4, 95% CI 1.1–2.0, and *p*-value 0.023) as the risk factors associated with the predisposition of rural children for getting STH (Table 4).

**Table 4:** Multivariate logistic regression for estimating the risk factors associated with increased STH in children

Risk factor	Category	AOR (95%CI)	*P*-value
Washing hands after defecating/before eating	No	7.0 (3.4–14.0)	< 0.001
House construction	Mud	3.5 (1.6–7.4)	0.001
Walking barefoot	Yes	2.2 (1.6–3.1)	< 0.001
Family size	≥ 5 people (overcrowded house)	1.6 (1.1–2.3)	0.023
Use of toilet	Open defecation	1.4 (1.1–2.0)	0.023
Consumption of raw vegetables	Yes	1.4 (0.9–2.2)	0.16
Habit of nail biting	Yes	1.0 (0.6–1.6)	0.999
Finger nails status	Untrimmed	0.3 (0.1–0.6)	0.002

*AOR* adjusted odds ratio, *CI* confidence interval, *STH* soil-transmitted helminth

## Discussion

In this community-based cross-sectional study, we studied 1426 rural children during a five-month period (June–October, 2020). The prevalence of STH among children of Daman district was 22.7%. Main risk factors associated with the predisposition of children for getting STH were not washing hands after defecating/before eating, living in mud house, walking barefoot, living in overcrowded house, and practicing open defecation.

In our study, prevalence of STH in children was 22.7%. This prevalence is more than the studies conducted in Nepal (3.1%) [20], Indonesia (10.1%) [21], and China (14.1%) [22]. Contrary, prevalence in our study is less than reported in studies from India (75.6%) [23], Ethiopia (51.5%) [24], Malaysia (37%) [25], and Tajikistan (32%) [26]. The prevalence differences observed in different parts of the world (and even different areas of the same country) are multifactorial; including variations in stool examination techniques, geographical location, time of study, type of study, age of study participants, culture, socio-economic status, literacy levels/occupations of the parents or guardians, food consumption habits, and personal hygiene behaviors. Table 5 compares the prevalence of STH infections among our study and two other studies conducted in Kandahar in 2003 and 2017. In our study, the prevalence of STH is lower but prevalence hookworm is higher than other two studies conducted in Kandahar. The STH prevalence of 22.7% in our study is might be underestimated, due to the fact that we collected only one sample of stool sample instead of the standard three samples (as the caretakers of the study participants were not very cooperative) and used the method of saline wet mount microscopy for the diagnosis of STH. On the other hand, decreased STH prevalence can be contributed to the Afghanistan ministry of public health implementation of mass deworming interventions by among children throughout Afghanistan.

**Table 5:** Prevalence of STH infections in Kandahar, in 2003, 2017, and 2020

	**2003** [18]	**2017** [19]	**2020 (this study)**
Any STH	42.8	46.8	22.7
*Ascaris lumbricoides*	37.4	45.5	18.7
Hookworm	0	0.5	7.5
*Trichuris trichiura*	7.8	1.4	1.4

In our study, most prevalent STH was *A. lumbricoides* (18.7%). The *A. lumbricoides* as the most common STH has also been reported in studies from Nepal (26.6%) [27], India (69.6%) [23], and Nigeria (75.6% [28]). A study was conducted on 207 adults and 179 children visiting health facilities of Ghazni and Parwan provinces of Afghanistan. This study concluded that the most prevalent STH among children was *A. lumbricoides* (25.1% in Ghazni province and 10.8% in Parwan province) [15]. In 2003, a study conducted among school children of Kabul, Nangarhar, Farah, and Kandahar provinces of Afghanistan revealed that the most prevalent STH was *A. lumbricoides* with the prevalence of 41% (408/1001) in the school children of these four provinces of Afghanistan [18]. In 2017, another study conducted in Kabul, Balkh, Herat, Nangarhar and Kandahar provinces of Afghanistan showed that the most prevalent STH infection was the one with *A. lumbricoides* (25.7%) [19].

Our study showed that children who were not washing hand after defecation and before eating were having statistically significant STH. Protective effects of handwashing have also been reported from Iran [29], Uzbekistan [30], China [22], India [31] [32], Nepal [20], Lao [33], Indonesia [21], and Ethiopia [34].

In this study, living in mud house was a risk factor associated with increased STH infection in children. Similar results have been reported in other studies [23, 35]. It could be due to the reason that mud has the tendency to retain helminth ova even when it has been apparently cleaned [36].

Our study revealed that walking barefoot is a risk factor for having increased STH infection. Similar results have been reported from Nepal [37], Thailand [38], Indonesia [21], Malawi [39], Ethiopia [40], and Kenya [41]. Walking barefoot is especially a risk factor for hookworms, as their larvae in the soil can penetrate into unbroken skin. Although walking barefoot is not directly related to infections of other helminths, but it indirectly leads to the infection when child touches the contaminated feet and eat with unwashed hands afterwards [36].

Practicing open defecation was also a risk factor of increased STH infection in our study. This result is in accordance with researches reported from India [42], Indonesia [21], Vietnam [43], Nigeria [44], and Kenya [41]. The ova of STH are present in contaminated human feces. So open defecation and poor sanitation helps in the direct contamination of soil and easily spread of the STH infection from child to child. Poverty was the main cause of open defection in our study participants as they were not having toilets at their houses.

## Limitations

There were some limitations in our study. We obtained only one fecal sample instead of the ideal three consecutive samples due to the level of cooperation and response of the parents and guardians. This might underestimate the real burden of STH. We did not find the intensity of STH in the study population. The only diagnostic method of saline wet mount microscopy used in our study has low sensitivity for the detection of STH infections. This was due to the fact that this was the only method available in the laboratories of our health facilities. Kato-Katz technique is not available in our laboratories. We did not get data of clinical symptoms and underlying diseases of the children, which can be confounding factors for STH. Additional studies should be performed in different parts of the country, including urban slums.

## Conclusion

Based on the results of our study, it can be concluded that the prevalence of STH infection among children in Daman district of Kandahar province is 22.7%. Main risk factors associated with the predisposition of rural children for getting STH were not washing hands after defecating/before eating, living in mud house, walking barefoot, living in overcrowded house, and practicing open defecation.

We recommend that improvement of socioeconomic status, sanitation, and health education to promote public awareness about health and hygiene together with periodic mass deworming programs are better strategies for the control of STH infections in Afghanistan. Meanwhile, government and international donor agencies in Afghanistan should help in improving socio-economic status of the rural areas through provision of basic facilities such as piped water, electricity, good housing, and proper toilets.

## Data Availability

All the data and materials related to this study are available on request.

## Abbreviations

*A. lumbricoides*: *Ascaris lumbricoides*
AOR: Adjusted odds ratio
CI: Confidence interval
COR: Crude odds ratio
SD: Standard deviation
STH: Soil-transmitted helminth
WHO: World Health Organization
