# Correction to: Prevalence and associated risk factors of soil-transmitted helminth infections in Kandahar, Afghanistan

**DOI:** 10.1186/s12879-022-07401-7

**Published:** 2022-04-28

**Authors:** Bilal Ahmad Rahimi, Bashir Ahmad Mahboobi, Mohammad Hashim Wafa, Mohammad Sediq Sahrai, Muhammad Haroon Stanikzai, Walter R. Taylor

**Affiliations:** 1 Department of Pediatrics, Faculty of Medicine, Kandahar University, Beside Aino Mena Town, District 10, Durahi, Kandahar, Afghanistan; 2 Head of Research Unit, Faculty of Medicine, Kandahar University, Kandahar, Afghanistan; 3 Department of Psychiatry, Faculty of Medicine, Kandahar University, Kandahar, Afghanistan; 4 Department of Internal Medicine, Faculty of Medicine, Kandahar University, Kandahar, Afghanistan; 5 Department of Public Health, Faculty of Medicine, Kandahar University, Kandahar, Afghanistan; 6 Mahidol Oxford Tropical Medicine Clinical Research Unit (MORU), Mahidol University, Bangkok, Thailand

## Correction to: BMC Infectious Diseases (2022) 22:361 10.1186/s12879-022-07336-z

Following publication of the original article [1], an error was identified in the Funding section.

The current statement reads:

There were no fundings for this study.

The statement should read:

This study did not receive any specific funding. WR Taylor is part funded by Wellcome under Grant 220,211. For the purpose of Open Access, the author has applied a CC BY public copyright licence to any Author Accepted Manuscript version arising from this submission.

The original article has been [1] updated.
